# Light, but Not Nutrients, Drives Seasonal Congruence of Taxonomic and Functional Diversity of Phytoplankton in a Eutrophic Highland Lake in China

**DOI:** 10.3389/fpls.2020.00179

**Published:** 2020-03-05

**Authors:** Huan Wang, Dandan Zhao, Liang Chen, John P. Giesy, Weizhen Zhang, Changbo Yuan, Leyi Ni, Hong Shen, Ping Xie

**Affiliations:** ^1^ Donghu Experimental Station of Lake Ecosystems, State Key Laboratory of Freshwater Ecology and Biotechnology, Institute of Hydrobiology, Chinese Academy of Sciences, Wuhan, China; ^2^ Guangzhou Chengyi Aquaculture Co., Ltd., Guangzhou, China; ^3^ State Key Laboratory of Eco-hydraulics in Northwest Arid Region, Faculty of Water Resources and Hydroelectric Engineering, Xi'an University of Technology, Xi'an, China; ^4^ Department of Veterinary Biomedical Sciences and Toxicology Centre, University of Saskatchewan, Saskatoon, SK, Canada; ^5^ Department of Environmental Science, Baylor University, Waco, TX, United States; ^6^ State Key Laboratory of Lake Science and Environment, Nanjing Institute of Geography and Limnology, Chinese Academy of Sciences, Nanjing, China; ^7^ State Key Laboratory of Plateau Ecology and Agriculture, Qinghai University, Xining, China

**Keywords:** seasonal succession, environmental drivers, algal taxonomic and functional groupings, alpha and beta diversity, eutrophication

## Abstract

Information on temporal dynamics of phytoplankton communities and their responses to environmental factors can provide insights into mechanisms driving succession of phytoplankton communities that is useful in programs to manage and or remediate undesirable assemblages. Populations of phytoplankton can be controlled by bottom-up factors such as nutrients and temperature or top-down such as predation by zooplankton. Traditionally, taxonomic diversity based on morphologies has been the measure used for analysis of responses to environmental factors. Recently, according to functional groupings, including functional groups (FG), morpho-FG (MFG), and morphology-based FG (MBFG), functional diversity has been used to represent functional aspects of phytoplankton communities. However, to what extent these taxonomic and functional groupings are congruent at seasonal time-scales and the main environmental factors, which drive succession, have remained less studied. Here, we analyzed absolute and relative proportions of a phytoplankton community during a 3-year period in Lake Erhai, a eutrophic highland lake in China. Alpha diversity and beta diversity, as measured by Shannon-Wiener and Bray-Curtis indices of taxonomic grouping and three functional groupings (FG, MFG, and MBFG) were applied to investigate environmental factors determining diversity. Significant, positive relationships were observed between taxonomic diversity and functional diversity that were strongly linked through seasons. In order to exclude the influence of dominant species' tolerance to extreme environments, the dominant species were excluded one by one, and the results showed that residual communities still exhibited similar patterns of succession. This synchronous temporal pattern was not principally driven by the dominant genera (*Microcystis*, *Psephonema*, and *Mougeotia*). Instead, the entire phytoplankton community assemblages were important in the pattern. Most diversity indices of taxonomic and functional groupings were significantly correlated with solar irradiance, but not nutrient concentrations. Because the lake is eutrophic and there were already sufficient nutrients available, additional nutrients had little effect on seasonal taxonomic and functional diversity of phytoplankton in Lake Erhai.

## Introduction

Phytoplankton, including planktonic algae and cyanobacteria, are primary producers in aquatic ecosystems, which play key roles in providing food for and affecting other organisms, and in turn are regulated by interactions with other organisms (Hutchins and Boyd, 2016; Kohlbach et al., 2016). Due to their small sizes, short life cycles of individual taxa and rapidly changing community structures, successional changes in phytoplankton communities are useful, rapid, integrative indicators of ecosystem status and trends in aquatic systems (Camp et al., 2015; Zwart et al., 2015). Compositions of species in algal communities are widely used to quantify temporal fluctuations and succession of aquatic ecosystems (Rodrigues et al., 2015; Wu et al., 2017; Cupertino et al., 2019). Succession of phytoplankton is related to multiple environmental factors such as nutrient concentrations, temperature, and quantity and quality of light in aquatic systems (Malik and Saros, 2016; Paerl et al., 2016; Thomas et al., 2017). It has long been debated whether numbers and types of phytoplankton are controlled by bottom-up factors, such as absolute and relative concentrations of nutrients, including phosphorus (P) or nitrogen (N), or by top-down processes, such as predation by zooplankton and fishes (Vanni et al., 1990; Xie and Liu, 2001; Zhang et al., 2008). Understanding mechanisms of phytoplankton succession and investigating responses in species composition to environmental factors can improve predictive power for phytoplankton responses to environmental changes.

Phytoplankton have been classified by kingdom, phylum, class, order, family, genus and species based on morphological characteristics and or pigments (Hu and Wei, 2006; Reynolds, 2006). In aquatic ecology, algal taxonomic variation has been used for explaining community properties and environmental variation (Lehtinen et al., 2017; Rozema et al., 2017). However, various species of phytoplankton in the same taxonomic association might have different morphological or functional features and taxonomically different algae usually co-occur in the same habitats (Reynolds et al., 2002; Salmaso and Padisák, 2007; Padisák et al., 2009; Kruk et al., 2010). Different phytoplankton taxa living in similar habitats often have the same ecological functions considering functional, morphological, physiological and ecological traits (Reynolds et al., 2002; Salmaso and Padisák, 2007; Padisák et al., 2009; Kruk et al., 2010). Thus, classifications of phytoplankton into functional groupings have been proposed, because functional and ecological traits can reflect strong mechanisms of natural selection (Salmaso et al., 2015; Özkundakci et al., 2016; Fontana et al., 2018). These include functional groups (FG, **Table 1**) (Reynolds et al., 2002; Padisák et al., 2009), morpho-FG (MFG) (Salmaso and Padisák, 2007) and morphology-based FG (MBFG) (Kruk et al., 2010). FG represents the classical and the widest used system of classifying phytoplankton, based on habitat properties, environmental tolerance and trophic state. FG integrates a base of information and relies on expert judgment (Hu et al., 2013; Zhu et al., 2013). MFG is identified using *a priori* determined traits influencing functional processes and ecological characteristics (Naselli-Flores et al., 2007; Mihaljević et al., 2013; Deng et al., 2019). MBFG uses exclusively morphological/structural criteria in the definition of groups and MBFG is an easier, but more effective classification that has only seven categories (Petar et al., 2014; Allende et al., 2019). Functional groupings with special traits can directly describe ecological processes, such as growth, sedimentation, grazing losses and nutrient acquisition (Weithoff, 2003). Functional groupings have been used as powerful and complementary approaches to describe dynamics of phytoplankton community assemblies and their ecological functions. Each of these methods has advantages and limitations.

**Table 1:** Comparisons of taxonomic and three functional groupings, including functional groups (FG), morpho-FG (MFG), and morphology-based FG (MBFG).

Taxonomic and functional groupings	Number of groups	Main grouping criteria	Principle of subdivision	Applied Cases
**Genus [based on (** **Hu and Wei, 2006**)]	Not Applicable	Phylogenetic characteristics	Size, population/single cell, color, structure and other features that can be observed under a microscope	Winder et al., 2008; Wang et al., 2010; Yang et al., 2012; Wu et al., 2013
**Functional groups (FG, based on** Reynolds et al., 2002 **and** Padisák et al., 2009)	39	Habitat, tolerances and sensitivities	Nutrient levels, water depth, salt and fresh water, scour, stratification, pH, transparency, light intensity and grazing	Vinebrooke et al., 2004; Fonseca and Bicudo, 2008; Stanković et al., 2012; Zhu et al., 2013; Borics et al., 2016
**Morpho-Functional Groups (MFG) [based on ** (**Salmaso and Padisák, 2007**)]	11 categories and 32 subcategories	Morphological and functional characteristics	size and form, mobility, potential mixotrophy, nutrient requirements, presence of gelatinous envelopes	Naselli-Flores et al., 2007; Mihaljević et al., 2013; Deng et al., 2019
**Morphologically based functional groups (MBFG) [based on ** (**Kruk et al., 2010**)]	7	Morphological and structural characteristics	Size, flagella, siliceous structures, mucilage, aerotopes and surface/volume ratio	Petar et al., 2014; Beamud et al., 2015; Mihaljević et al., 2015; Bortolini et al., 2016; Kruk et al., 2017; Cupertino et al., 2019

Trait-based groupings have identified environmental factors that determine succession of phytoplankton communities in temperate (Weithoff et al., 2015), tropical (Costa et al., 2009; Rodrigues et al., 2018), subtropical (Becker et al., 2009) and Mediterranean (Becker et al., 2010) regions, such as the environmental resources, environmental change and predation (Reynolds, 1984; Salmaso and Padisák, 2007; Salmaso et al., 2015). Results of previous studies have shown that composition, biomass and diversity of phytoplankton is primarily determined by nutrients (Reynolds and Irish, 1997; Rangel et al., 2012). Results of other studies have shown that succession of phytoplankton communities is controlled by physical conditions such as light (Edwards et al., 2015), water temperature (Rasconi et al., 2017; Neukermans et al., 2018), mean depth (Pinckney et al., 2015), flushing rate (Cañavate et al., 2015) and their interactions (Hart et al., 2015; Edwards et al., 2016; Burson et al., 2018; Richardson et al., 2019). Competition for resources, predation, environmental change and rates of mutation and plasticity will affect succession of taxonomic and functional groupings, but whether there is seasonal congruence between functional and taxonomic groupings or not remains unclear in natural communities. If the answer is yes, then, how is this congruence driven by environmental factors?

Diversity indices, of algal taxonomic and functional groupings, such as Shannon-Wiener or Bray-Curtis, have been used to track succession of communities in response to environmental factors (Maloufi et al., 2016; Vallina et al., 2017). Diversity of phytoplankton communities based on taxonomy of algae can be used to describe patterns of succession and have been used to analyze the ecological status of assemblages of phytoplankton (Rodrigues et al., 2015; Wojciechowski et al., 2017). Functional diversity of algae is based mainly on similarities of morpho-functional traits between species and are directly related to environmental factors, therefore, functional diversity affects processes at all scales of community and ecosystem organization (Zwart et al., 2015; Vallina et al., 2017). Therefore, taxonomic plus functional classifications might be a useful combination to assess environmental factors influencing aquatic communities (Cupertino et al., 2019). It is critical to understand how various factors are coupled if ecosystems are to be managed and/or restored to provide particular ecological services for humans.

In this study, data from a 36-month study of phytoplankton in Lake Erhai, a eutrophic highland lake in China, was used to calculate diversity indices of taxonomic and three functional groupings, including FG, MFG, and MBFG. Three hypotheses were tested: (1) taxonomic and functional diversity are positively correlated and their diversity indices show the same synchronous seasonal patterns; (2) seasonal congruence is generally driven by one or a few dominant genera. In eutrophic systems, the genera *Microcystis, Psephonema*, *and Mougeotia*, which are adapted to conditions of greater concentrations of phosphorus, can determine the temporal pattern of functional and taxonomic groupings; and (3) seasonal congruence is caused by the fact that functional and taxonomic diversity respond in a similar way to environmental factors. To test for seasonal congruence and environmental drivers of phytoplankton taxonomic and functional groupings, in the present study, results of which are reported here, both alpha (α) and beta (β) diversities of genera grouping and three functional groupings (FG, MFG, and MBFG) of phytoplankton with monthly measurements were compared. In addition, to test if dominant genera were most important determinants of changes in composition of species, dominant genera, were sequentially removed and diversity indices were recalculated to determine if removing these genera would affect the seasonal patterns.

## Methods

### Study Site and Sampling Method

**Figure 1:**
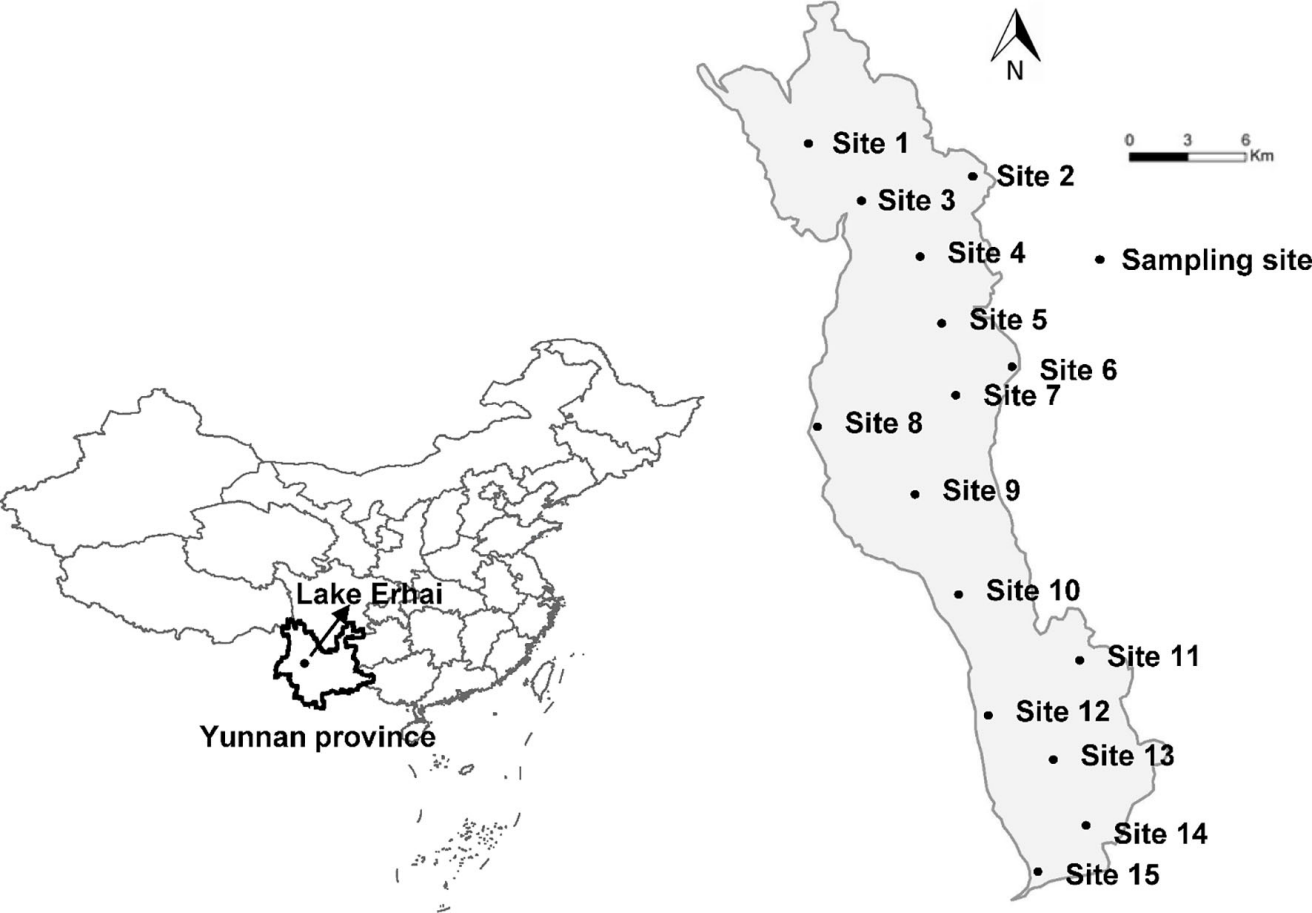
Map of Lake Erhai and the location of the 15 sampling sites.

The study was performed in Lake Erhai (25°36′-25°58′ N, 100°05′-100°17′ E), the second largest, high-altitude freshwater lake on the Yunnan Plateau, China (**Figure 1**). Lake Erhai is located in the central zone of the Dali Bai Autonomous Prefecture in Yunnan Province. Lake Erhai has a total surface area of approximately 250 km^2^, an elevation of 1,974 m and a volume of nearly 28.8 × 10^8^ m^3^. Mean and maximum depth are 10.5 and 20.5 m, respectively. Lake Erhai, is currently in the early stages of eutrophication (Lin et al., 2016; Wang et al., 2018), with concentration of total nitrogen (TN) of 0.7 mg/L (Zhu et al., 2018), total phosphorus (TP) of 0.03 mg/L (Zhu et al., 2018) and chlorophyll a (Chl a) of 13.33 µg/L during June 2013 to May 2015, with a peak value exceeding 30 µg/L (Wang et al., 2018), all of which exceed the threshold value of the eutrophication categories (TP > 0.03 mg/L, TN > 0.65 mg/L, and Chl *a* > 9 µg/L, Nürnberg, 1996). Samples were collected monthly, from 15 locations, between January 2012 and December 2014 (**Figure 1**). Composite, integrated samples were collected by combining samples of water from the upper (0.5 m below the water surface), middle (midway between the surface and the bottom), and lower (0.5 m above the sediment surface) portions of the water column at each site. Composite, integrated samples were used for analysis of nutrient concentration and phytoplankton.

### Physical and Chemical Analysis

Water temperature (T) was measured at 0.5 m below the water surface at each sampling site using a YSI ProPlus multiparameter water quality meter (Yellow Springs, OH, USA). Water transparency (SD) was measured using a Secchi disk (20 cm diameter) *in situ*. Secchi depth is a visual measure of light intensity, because light is absorbed by particles and soluble substances in the water, resulting in disappearance of view at a certain depth (Secchi, 1866; Preisendorfer, 1986). Concentrations of dissolved inorganic phosphorus (DIP) and ammonium (NH_4_-N) in mixed samples were measured by use of standard preservation and analytical procedures established by Government Water Association (APHA-AWWA-WPCF, 1915).

### Identification and Enumeration of Phytoplankton

One-liter samples were fixed with 10 ml Lugol's iodine solution and were concentrated to 50 ml with a siphon after sedimentation for 48 h in Utermhol chambers (Huang et al., 1999). After mixing, concentrated samples (0.1 ml) were observed with a phytoplankton-counting chamber (0.1 ml, Institute of Hydrobiology, Chinese Academy of Sciences, China) under 400× magnification using a light microscope (Olympus BX21, Tokyo, Japan). Cells of colonial or filamentous (e.g., *Microcystis*, *Psephonema*, *Mougeotia*, and *Oscillatoria*) algae were separated using an ultrasonic device (JY88-II, Scientiz, Ningbo, Zhejiang, China) before enumeration. Taxonomic identification of phytoplankton was performed according to Hu and Wei (Hu and Wei, 2006). Species were aggregated into kingdom, phylum, class, order, family, genus and species (if needed) levels.

### Sorting of Functional Groupings

Three functional classification schemes, including FG, MFG, and MBFG, were performed. FG, based on genera or species, when possible, resulted in 39 groups (Reynolds et al., 2002), identified by use of alpha-numeric codes according to their similar morphology, environmental sensitivity and tolerance, based on Grime's (1979) seminal work on terrestrial vegetation, which were applied to phytoplankton. MFG was composed of 32 groups, based on motility, the potential capacity to obtain carbon and nutrients by mixotrophy, specific nutrient requirements, size and shape, and presence of gelatinous envelopes (Salmaso and Padisák, 2007). MBFG was composed of 7 groups based on eight morphological traits of phytoplankton, including flagella, mucilage, siliceous exoskeletal structures, aerotopes, gas vesicles, volume, surface/volume, and maximum linear dimension characterized by use of light microscopy (Kruk et al., 2010).

### Statistical Analyses

Indices of α diversity, calculated by use of the Shannon-Wiener index (Shannon, 1948), and β diversity, calculated by use of the Bray-Curtis dissimilarity index (Bray and Curtis, 1957), were applied to characterize taxonomic groupings and three functional groupings, including FG, MFG, and MBFG. The Shannon-Wiener index of each taxonomic and functional classification in each site for each month was calculated. The Bray-Curtis index was calculated to measure pairwise dissimilarity in species composition between sites within each month. Mean pairwise dissimilarity among sites for each month was used as a response variable, i.e. spatial β diversity within the lake. Both Shannon-Wiener and Bray-Curtis indices of each taxonomic and functional classification were calculated in the same way, with serial removal of the three most dominant genera, which were taxa accounting for more than 70% of total cell numbers. To investigate seasonality of environmental parameters and diversity indices, a locally weighted scatter smoothing function, using month as the predictor variable (Cleveland, 1981), was used to fit curves (span = 0.75) for these variables. To test if taxonomic and functional diversity indices were related, generalized linear mixed models [GLMMs (Bolker et al., 2009)] were used with pairs of mean diversity indices for each month from all sampling points as variables. To test how environmental factors affect taxonomic and functional diversity indices and drive the congruence effect, in the GLMMs, the mean values of each taxonomic and functional diversity index in the 15 sites for each month were used as response variables, and DIP, NH_4_-N, T, and SD were used as predictors. To avoid pseudo-replication (pseudo-replication typically occurs when the number of observations or the number of data points is treated inappropriately as independent replicates) in the analysis of the correlation among diversity indices, a seasonal (month) effect was introduced as a random predictor variable (Hurlbert, 1984). All statistical analyses were conducted in R 3.1.0 (R Development Core Team, 2014).

## Results

**Figure 2:**
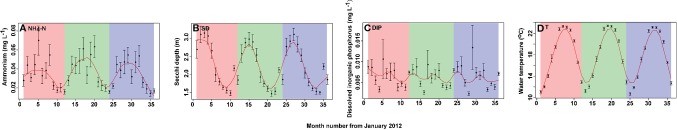
Monthly time series of values of physical and chemical parameters in Lake Erhai from January 2012 to December 2014. **(A)** ammonium (NH_4_-N), **(B)** Secchi depth (SD), **(C)** dissolved inorganic phosphorus (DIP), and **(D)** water temperature (T). Values are presented as the mean ± standard deviation (SD) among the 15 sites for each month. To detect the seasonality of environmental parameters, a locally weighted scatter smoothing function (Cleveland, 1981) was used to fit a smooth curve (span = 0.75) using month as the predictor variable.

Mean values of concentrations of nutrients, including NH_4_-N and DIP, and physical parameters, including Secchi depth (SD) and water temperature (T), of the 15 sampling sites from January 2012 to December 2014 are shown (**Figure 2**). All parameters exhibited seasonal variations. Concentrations of NH_4_-N were greatest during summer, while concentrations of DIP were greater during spring and autumn, SD was maximum in spring and T reached its maximum in August. Mean concentrations of NH_4_-N, DIP, SD, and T were 0.039 mg/L, 0.006 mg/L, 2.22 m, and 18.12℃, respectively.

**Figure 3:**
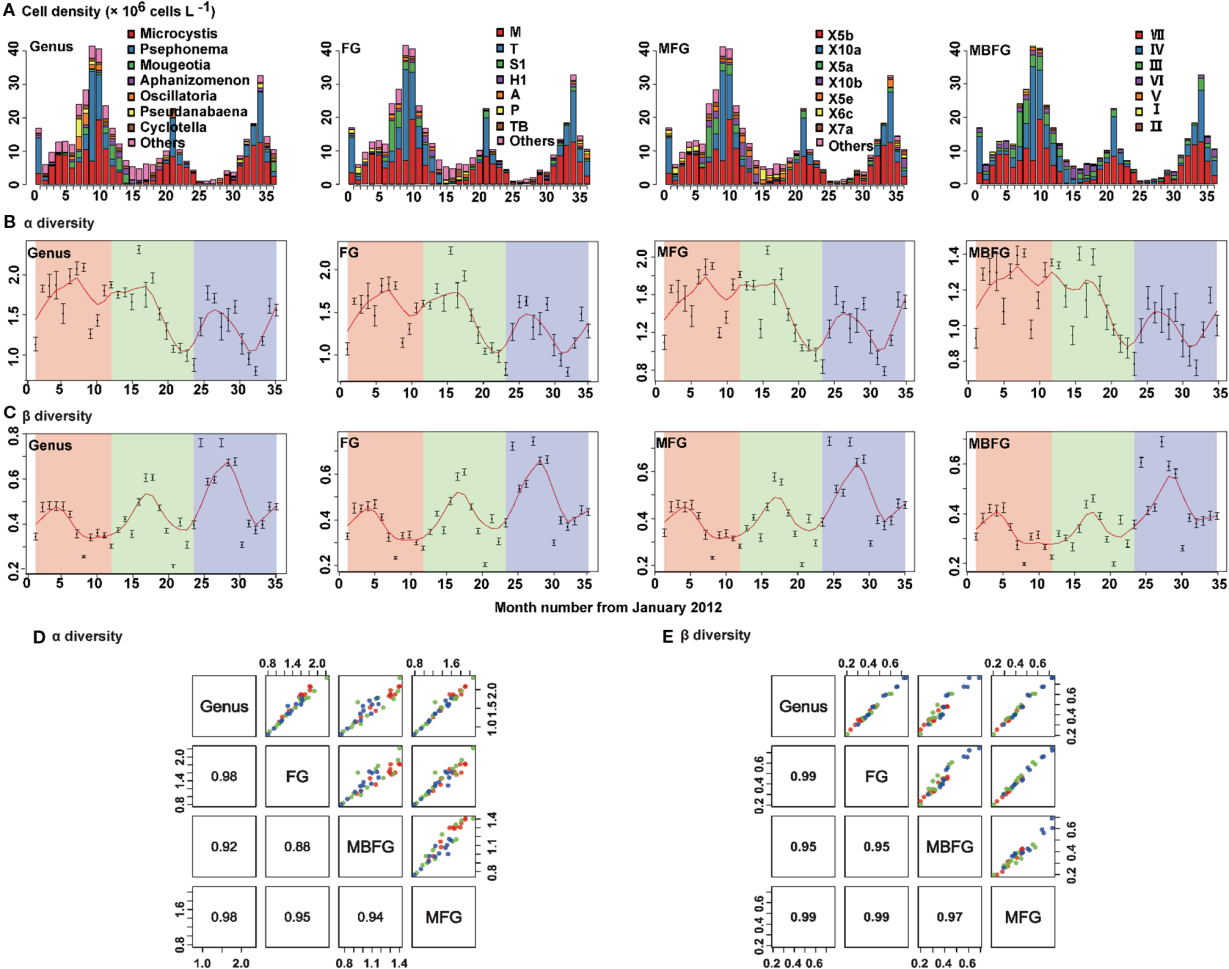
Seasonal composition and successions of phytoplankton in Lake Erhai from January 2012 to December 2014 according to taxonomic and three functional groupings. **(A)** Cell density and composition, **(B)** alpha diversity (Shannon-Wiener diversity) indices, **(C)** beta diversity (Bray-Curtis dissimilarity) indices, **(D)** pair plot for linear fitting of alpha and **(E)** beta diversity. The order from left to right is genus, functional groups (FG), morpho-FG (MFG), and morphology-based FG (MBFG). The cell density values are presented as the mean of 15 sites for each month. Alpha and beta diversity values are presented as the mean ± standard deviation (SD) among the 15 sites for each month. To detect the seasonality of alpha and beta diversity indices, a locally weighted scatter smoothing function (Cleveland, 1981) was used to fit a smooth curve (span = 0.75) using month as the predictor variable. The numbers in the lower left columns in (d) and (e) are correlation coefficients (R^2^) (*p* < 0.05).

Patterns of succession of compositions of species in taxonomic and functional groupings of phytoplankton communities during the sampling period are shown (**Figure 3**). Cyanophyta, Chlorophyta and Bacillariophyta were the three dominant phyla. Cyanophyta was the dominant phylum from July to December (blooming period), whereas Bacillariophyta dominated from February to June. Populations of Chlorophyta peaked during summer and then decreased from summer to winter. Communities of cyanophyta were by the genera *Microcystis* and *Aphanizomenon,* and Chlorophyta was dominated by genera *Psephonema* and *Mougeotia* (**Figure 3A**-Genus). Thirty-two (32) FG groups were identified, with the primary three being M dominated by *Microcystis*, T dominated by *Psephonema*, and S1 dominated by *Oscillatoria* (**Figure 3A**-FG). A total of 23 MFG groups were identified with the primary three being X5b, dominated by *Microcystis*, X10a, dominated by *Psephonema*, and X5a, dominated by *Oscillatoria and Aphanizomenon* (**Figure 3A**-MFG). Seven (7) MBFG groups were identified, with the three primary being VII, dominated by *Microcysti*s, IV, dominated by *Psephonema* and III, dominated by *Oscillatoria* (**Figure 3A**-MBFG).

For both α and β diversities, taxonomic (genus) and functional diversities of FG, MFG, and MBFG exhibited maxima during summer then decreased in autumn (**Figures 3B, C**). Significant positive correlations were also observed between taxonomic and functional groupings (**Figures 3D, E**). The coefficients of determination (R^2^) between taxonomic and functional α diversities were ≥ 0.88 (*p* < 0.05) (**Figure 3D**) and the R^2^ of β diversities were ≥ 0.95 (*p* < 0.05) (**Figure 3E**).

The dominant genera, which accounted for > 70% of total numbers of cells, were *Microcystis*, *Psephonema*, and *Mougeotia* (**Figure S1**). To check whether dominant genera affected seasonal patterns of diversity indices, these three dominant genera were removed serially. Successional patterns of taxonomic and functional groupings of the rest of the phytoplankton community are shown in **Figures S2A**, **S3A**, and **S4A** (supplementary information). The α (**Figures S2B**, **S3B**, and **S4B**) and β diversity (**Figures S2C**, **S3C**, and **S4C**) indices of the remaining phytoplankton community showed a similar seasonal pattern to those of the whole community, and significant correlations (*p* < 0.001) were found for genus, FG, MFG, and MBFG groupings (**Figure S5**).

Results of GLMMs showed that both α and β diversities of taxonomic and functional groupings responded similarly to environmental factors (**Table 2**). Except for α diversity of MFG and MBFG groupings, all the other diversity indices were significantly correlated with SD (*p* < 0.05) (**Table 2**). Meanwhile, β diversity of FG was significantly correlated with water temperature (*p* < 0.05) (**Table 2**). However, all diversities showed weaker correlations with concentrations of NH_4_-N and DIP than with SD or water temperature.

**Table 2:** Generalized linear mixed models (GLMMs) results for environmental factors and diversity indices of taxonomic and three functional groupings, including functional groups (FG), morpho-FG (MFG), and morphology-based FG (MBFG).

	Group	(Intercept)	Ammonium	Phosphorus	Secchi depth	Water temperature	rvalue
Alpha diversity (Shannon-Wiener index)	Genus	0.851	0.65	0.652	**0.029**	0.226	0.244
	FG	0.668	0.723	0.7	**0.01**	0.115	0.322
	MFG	0.847	0.563	0.473	0.082	0.311	0.15
	MBFG	0.409	0.615	0.771	0.1	0.251	0.122
Beta diversity (Bray-Curtis index)	Genus	0.173	0.161	0.098	**0.002**	0.053	0.357
	FG	0.156	0.192	0.112	**0.003**	**0.047**	0.346
	MFG	0.183	0.121	0.073	**0.003**	0.051	0.334
	MBFG	0.246	0.143	0.131	**0.007**	0.053	0.255

The bolded numbers showed correlation coefficients (R2) with statistical significance (p < 0.05).

## Discussion

In Lake Erhai, taxonomic and functional diversities were positively correlated and both alpha and beta indices showed the same synchronous seasonal pattern. The maximum species diversity was observed under multiple conditions of nutrient concentrations, where nutrients and light provided ample scope for coexistence of species (Török et al., 2016), therefore both functional and taxonomic diversity increased during spring, followed by a decrease in summer and then increasing in winter. Results of previous studies have shown that seasonal congruence occurs when selection acts predominantly on one trait so that increase or decrease of species characterized by this trait will result in increase or decrease of both functional and taxonomic diversity (Weithoff et al., 2015). In this study, due to increases in light intensity, some groups, such as Bacillariophyta would be eliminated through competition because they could not adapt to strong light (Reynolds et al., 2002). However, other genera, such as *Microcystis* and *Psephonema* which are better adapted to the changed environment would outcompete (Reynolds et al., 2002). This seasonal congruence in phytoplankton species composition illustrate the changes in functional diversity in Lake Erhai and this algal succession is a distinguishable yearly cycle in many natural aquatic ecosystems (Lund, 1965; Reynolds, 2006). These results suggest that functional diversity may encompass the overall variability of taxonomic diversity. Interestingly, assemblage structure in simplified classifications (MBFG and MFG) was affected by the same environmental variation, further highlighting the similarity of taxonomic and functional groupings in this eutrophic highland lake.

During the entire study period, from 2012 to 2014, significant, positive correlations were observed between taxonomic and functional diversities. However, the seasonal congruence of taxonomic and functional diversities was not driven by the dominant genera, suggesting that the phytoplankton community consisted of species with similar adaptive strategies (Reynolds, 1984). Since coexisting species represented FG with different photosynthetic pigments, it was verified that niche differentiation in the light spectrum played a role. In eutrophic environments, adequate supply of nutrients promotes biomass growth (Agawin et al., 2000; Søndergaard et al., 2017), and the species interact through mutual shading, and the best light competitor (*Microcystis*) is expected to prevail, and results in blooms. Under the cover of *Microcystis*, conditions for growth of *Psephonema* and *Mougeotia* result in large numbers of those species. Light is considered especially important for *Microcystis* and FG M, X5b and VII (Reynolds et al., 2002; Salmaso and Padisák, 2007; Padisák et al., 2009; Kruk et al., 2010), which were most represented in Lake Erhai. Because the three dominant groups accounted for the majority of cell density (>70%), interferences of dominant species were excluded. However, the results were still significantly correlated, which suggested that responses of phytoplankton communities to the environment were not based on dominant effects of individual species.

Results of the study presented here revealed diversities of taxonomic and functional groupings responded in a similar way to light levels and there is no significant relationship between diversities and concentrations of nutrients. Results of previous studies showed that the seasonal pattern of diversity indices of both taxonomic and functional groupings is dependent on the environmental factors (Becker et al., 2010; Weithoff et al., 2015). The fundamental factors that determine algal seasonal diversity are the physico-chemical characteristics in the water column such as nutrient concentrations, water temperature and light (Smith, 1986; Reynolds, 1989; Jensen et al., 1994; Elliott, 2010). Understanding how environmental variations affect the biodiversity and succession of phytoplankton is a key challenge. Results of several studies have shown that composition and biomass of phytoplankton were shaped by nutrients in nutrient-poor environments (Alpine and Cloern, 1992; Watson et al., 1997). In oligotrophic environments, structures of algal communities are driven by strong competition for nutrients (Tsiola et al., 2016). Limitation by individual nutrients prevents accumulation of sufficient biomass of phytoplankton, so shading effects and competition for light are negligible. In addition, increasing nutrient loads caused changes in phytoplankton species composition by shifting the species interactions from competition for nutrients to competition for light (Burson et al., 2018). In this study, no significant correlation between nutrient concentrations and phytoplankton community structure were found over time in Erhai Lake. Instead, water transparency (SD) and water temperature were strongly related to seasonal succession of phytoplankton community, especially SD. Therefore, nutrient concentrations are not a constraint for phytoplankton in eutrophic Lake Erhai, with means of concentrations of 0.039 mg NH_4_-N/L, 0.006 mg DIP/L, respectively (Wen and Ma, 2011). This result is consistent with the Liebig Law of the Minimum, which states that only one factor, such as a nutrient, can limit a biological process, such as primary productivity of phytoplankton (Liebig, 1840). For instance, in natural systems, concentrations of phosphorus (P) are often limiting (Krom et al., 1991). However, after cultural eutrophication during which P is added due to human activities, such as agriculture or urbanization, once the minimum is exceeded, P is no longer limiting to primary production. Relative proportions of nitrogen (N) and P needed to support primary productivity is defined by the Redfield number, which is the ratio of N to P in cells (N/P ratio of 16) (Redfield, 1958). In North American lakes, succession of phytoplankton was determined not by predation by zooplankton or temperature or depletion of nutrients but rather sensitivities of species of phytoplankton to ultraviolet (UV) light (Gala and Giesy, 1991). Results of previous studies also showed that seasonal variation in phytoplankton community composition was affected by light (SD, water transparency and UV radiation) in eutrophic lakes (Sommaruga and Augustin, 2006; Wondie et al., 2007). In this study, light conditions, but not nutrients, is also the key driver of algal taxonomic and functional diversities in eutrophic Lake Erhai. Further studies might be required to explore how solar UV radiation and visible light affect the succession of phytoplankton in different lakes, especially in highland lakes with high intensities of light.

Since the MBFG approach is easier and less resource intensive than genus level and other functional classifications, it is suitable for routine biomonitoring, long-term studies, or to process large amounts of samples when comparing systems. Nonetheless, the genus-level taxonomic approach, FG and MFG provided the detailed information and multiple insights on assemblage dynamics of phytoplankton in this study, and using multiple classifications at the same time can provide the most detailed variation for further analysis.

## Conclusions

In Lake Erhai, a eutrophic highland lake in China, significant positive relationships were observed between taxonomic diversity and functional diversity of phytoplankton, with a strong synchronous seasonal pattern of succession. Taxonomic and functional diversity can complement each other and provide a more comprehensive explanation of the driving effect of environmental changes on phytoplankton communities from biological and functional perspectives. Results of this study demonstrated that functional groupings can be used as simple avenues for studying temporal patterns of phytoplankton community assembly and the environmental drivers in eutrophic lake. Furthermore, the seasonal congruence was not driven by the dominated genera, *Microcystis*, *Psephonema*, and *Mougeotia*, which suggested that the algal community consisted of species with similar ecological strategies. Both functional diversity and taxonomic diversity were significantly, positively correlated with light conditions, but not concentrations of nutrients. Conclusively, light is the key driver of seasonal congruence of phytoplankton taxonomic and functional diversity in this eutrophic highland lake. Our results also showed that alternative functional groupings of phytoplankton can be reliable predictors of environment-biological relationships.

## Data Availability Statement

All datasets generated for this study are included in the article/**Supplementary Material**.

## Author Contributions

PX and HS designed the study. HW and DZ conducted the experiments. HW led the data processing and manuscript writing. HS, LC and JG revised the manuscript. WZ and CY helped performance of data analysis. LN helped experimental process. All authors contributed to the final draft.

## Conflict of Interest

The authors declare that the research was conducted in the absence of any commercial or financial relationships that could be construed as a potential conflict of interest.
