# Supplementary material for: Light, but Not Nutrients, Drives Seasonal Congruence of Taxonomic and Functional Diversity of Phytoplankton in a Eutrophic Highland Lake in China
Source: Front Plant Sci. 2020 Mar 5;11:179. doi: 10.3389/fpls.2020.00179 (PMC7067047; doi:10.3389/fpls.2020.00179)
Supplement: Supplementary file 1 [file DataSheet_1.pdf]

Supplementary information

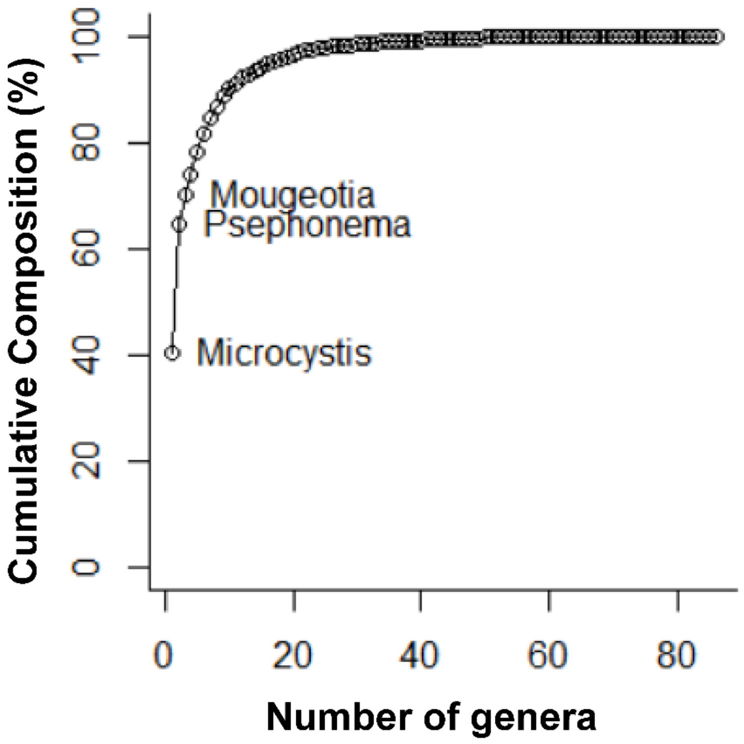

Figure S1. Accumulation curve of cell density of phytoplankton by all the genera. Genera are presented in descending order according to numbers of cells. Dominant genera, *Microcystis*, *Psephonema* and *Mougeotia* contributed more than 70% of the total numbers of cells.

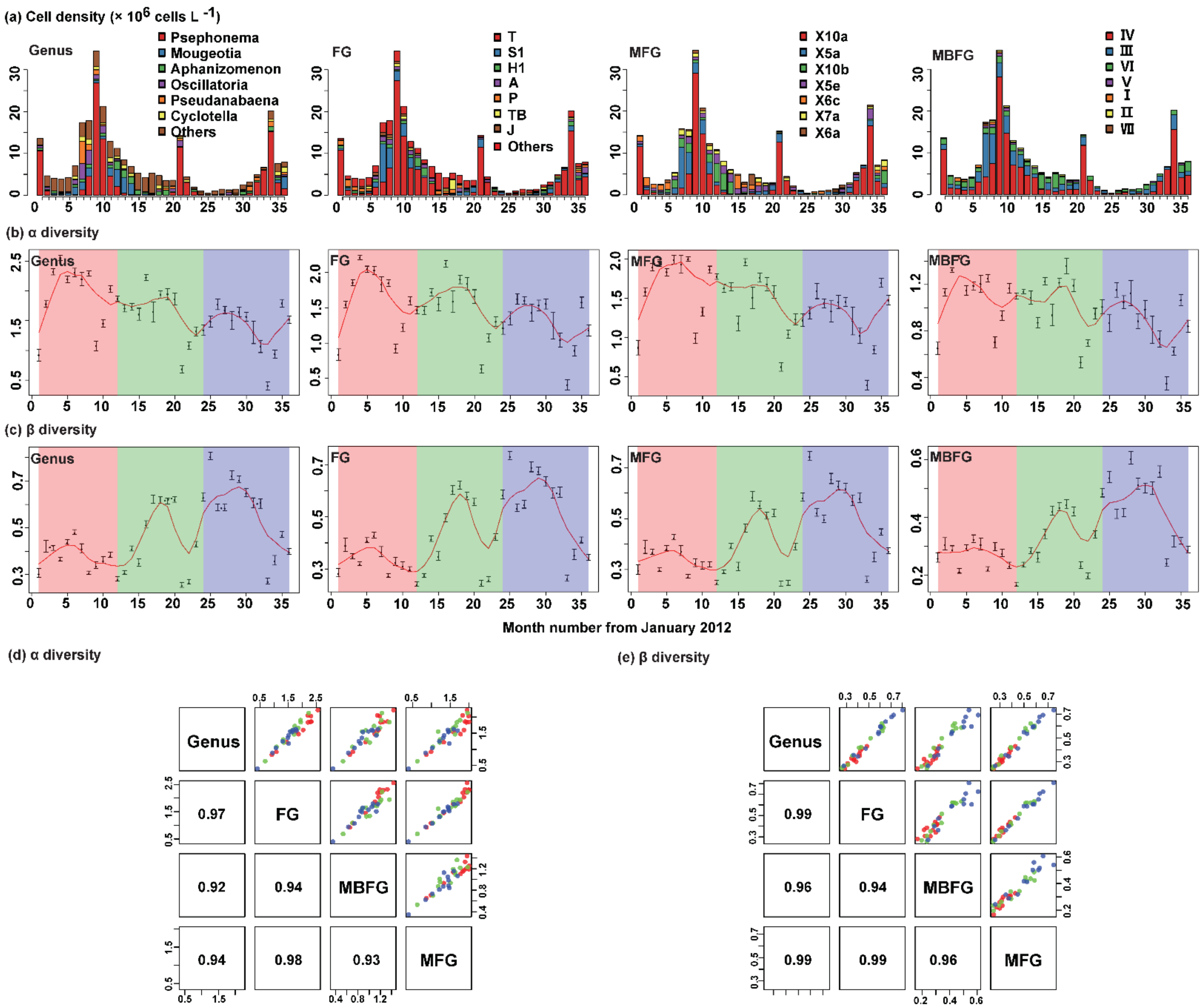
(a) Cell density (× 10^6 cells L^-1)
Genus
Psephonema
Mougeotia
Aphanizomenon
Oscillatoria
Pseudanabaena
Cyclotella
Others
FG
T
S1
H1
A
P
TB
J
Others
MFG
X10a
X5a
X10b
X5e
X6c
X7a
X6a
MBFG
IV
III
VI
V
I
II
VII
(b) α diversity
Genus
FG
MFG
MBFG
(c) β diversity
Genus
FG
MFG
MBFG
Month number from January 2012
(d) α diversity
Genus
0.97
FG
0.92
0.94
MBFG
0.94
0.98
0.93
MFG
(e) β diversity
Genus
0.99
FG
0.96
0.94
MBFG
0.99
0.99
0.96
MFG

Figure S2. Seasonal composition and successions of phytoplankton in Lake Erhai from January 2012 to December 2014 according to taxonomic and 3 functional groupings, with removal of the dominant genus *Microcystis*. (a) Cell density and composition, (b) alpha diversity (Shannon-Wiener diversity) indices, (c) beta diversity (Bray-Curtis dissimilarity) indices, (d) pair plot for linear fitting of alpha and (e) beta diversity. The order from left to right is genus, functional groups (FG), morpho-functional groups (MFG) and morphology-based functional groups (MBFG). Numbers of cells are presented as the mean of the 15 sites for each month. Alpha and beta diversity values are presented as the mean ± standard deviation (SD) among the 15 sites for each month. The numbers in the lower left columns in (d) and (e) are correlation coefficients (R^2) ($p <$ 0.05).

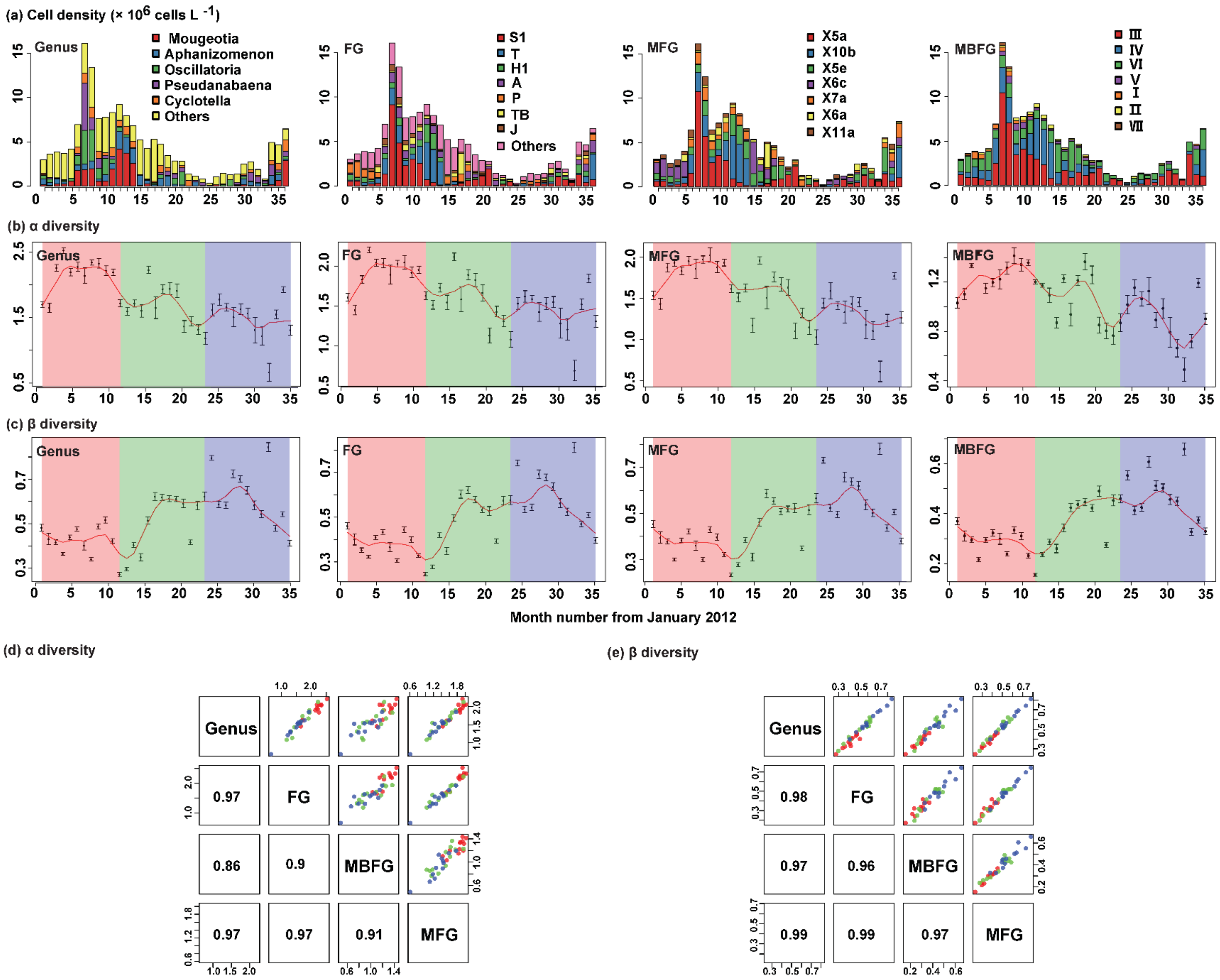
(a) Cell density (× 10^6 cells L^-1)
Genus
Mougeotia
Aphanizomenon
Oscillatoria
Pseudanabaena
Cyclotella
Others
FG
S1
T
H1
A
P
TB
J
Others
MFG
X5a
X10b
X5e
X6c
X7a
X6a
X11a
MBFG
III
IV
VI
V
I
II
VII
(b) α diversity
Genus
FG
MFG
MBFG
(c) β diversity
Genus
FG
MFG
MBFG
Month number from January 2012
(d) α diversity
Genus
FG
MBFG
MFG
0.97
0.86
0.9
0.97
0.97
0.91
(e) β diversity
Genus
FG
MBFG
MFG
0.98
0.97
0.96
0.99
0.99
0.97

Figure S3. Seasonal composition and successions of phytoplankton in Lake Erhai from January 2012 to December 2014 according to taxonomic and 3 functional groupings, with removal of the dominant genera *Microcystis* and *Psephonema*. (a) Cell density and composition, (b) alpha diversity (Shannon-Wiener diversity) indices, (c) beta diversity (Bray-Curtis dissimilarity) indices, (d) pair plot for linear fitting of alpha and (e) beta diversity. The order from left to right is genus, functional groups (FG), morpho-functional groups (MFG) and morphology-based functional groups (MBFG). Numbers of cells are presented as the mean of the 15 sites for each month. Alpha and beta diversity values are presented as the mean ± standard deviation (SD) among the 15 sites for each month. The numbers in the lower left columns in (d) and (e) are correlation coefficients (R^2) ($p < 0.05$).

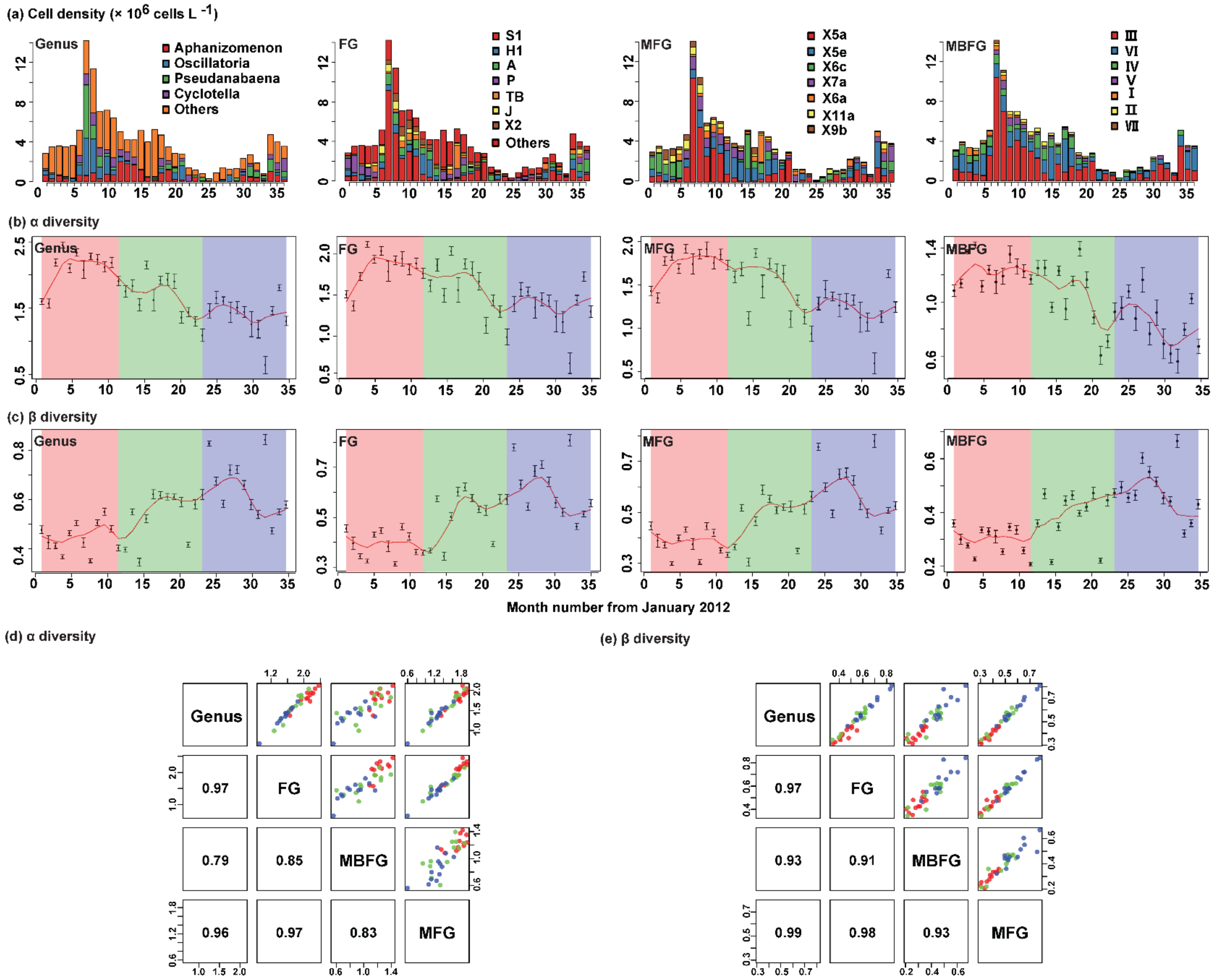
(a) Cell density (× 10^6 cells L^-1)
Genus
Aphanizomenon
Oscillatoria
Pseudanabaena
Cyclotella
Others
FG
S1
H1
A
P
TB
J
X2
Others
MFG
X5a
X5e
X6c
X7a
X6a
X11a
X9b
MBFG
III
VI
IV
V
I
II
VII
(b) α diversity
(c) β diversity
Month number from January 2012
(d) α diversity
Genus
FG
MBFG
MFG
0.97
0.79
0.85
0.96
0.97
0.83
(e) β diversity
Genus
FG
MBFG
MFG
0.97
0.93
0.91
0.99
0.98
0.93

Figure S4. Seasonal composition and successions of phytoplankton in Lake Erhai from January 2012 to December 2014 according to taxonomic and 3 functional groupings, with removal of the dominant genera *Microcystis*, *Psephonema* and *Mougeotia*. (a) Cell density and composition, (b) alpha diversity (Shannon-Wiener diversity) indices, (c) beta diversity (Bray-Curtis dissimilarity) indices, (d) pair plot for linear fitting of alpha and (e) beta diversity. The order from left to right is genus, functional groups (FG), morpho-functional groups (MFG) and morphology-based functional groups (MBFG). Numbers of cells are presented as the mean of the 15 sites for each month. Alpha and beta diversity values are presented as the mean ± standard deviation (SD) among the 15 sites for each month. The numbers in the lower left columns in (d) and (e) are correlation coefficients (R^2) ($p < 0.05$).

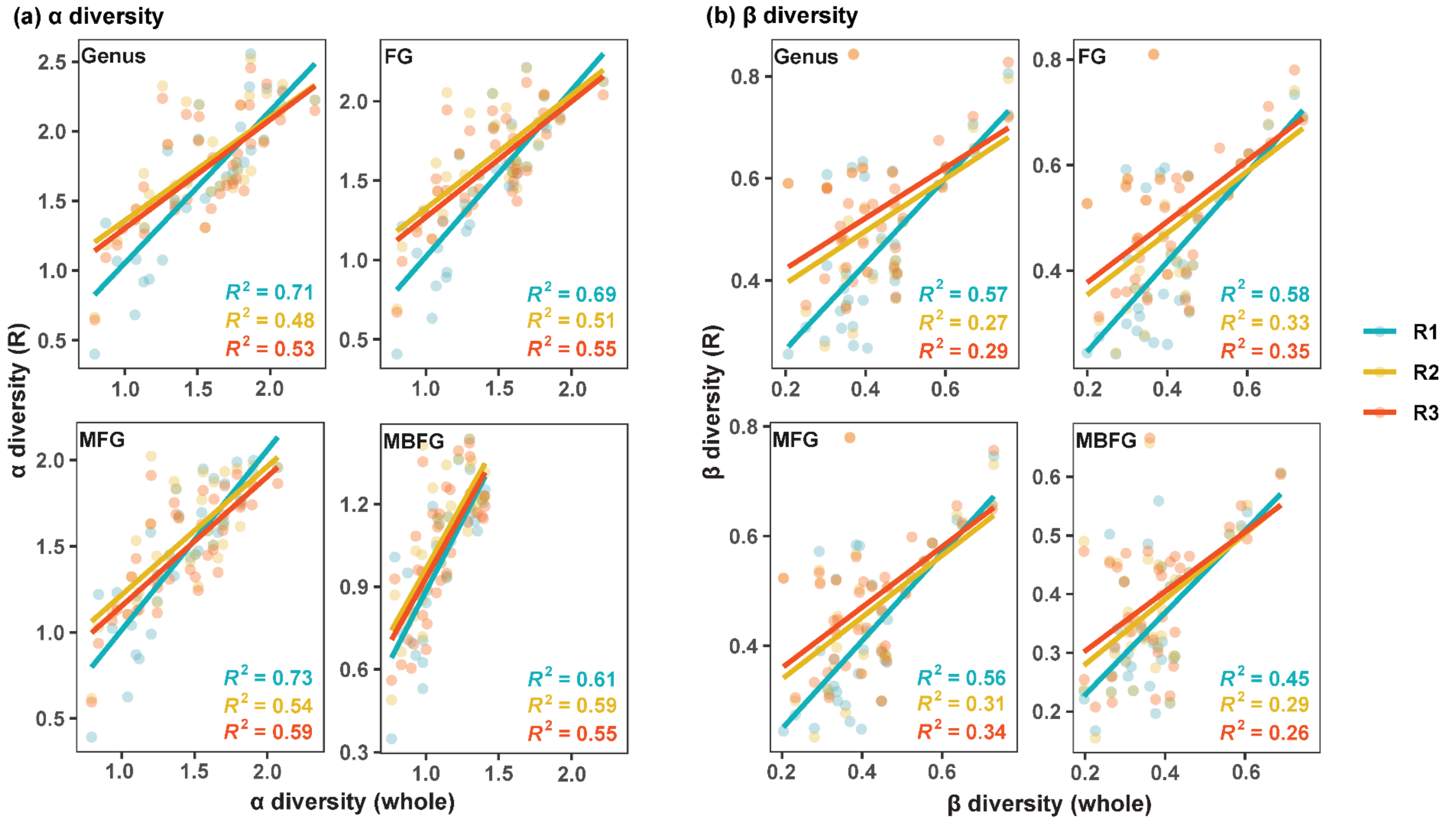

Figure S5. Correlation analyses presented as pair-wise scatter plots and linear regressions, for (a) α diversity (Shannon-Wiener) and (b) β diversity (Bray-Curtis) indices of genus between the whole community and the remaining community (R) after the dominant genera *Microcystis*,

Psephonema and *Mougeotia* were removed gradually. R1: removal of *Microcystis*, R2: removal of *Microcystis* and *Psephonema*, R3: removal of *Microcystis*, *Psephonema* and *Mougeotia*. The numbers in the lower right are correlation coefficients (R^2) ($p < 0.001$).
